# Predictive value of remnant cholesterol for in-stent restenosis after drug-eluting stent implantation

**DOI:** 10.1097/MD.0000000000050097

**Published:** 2026-08-07

**Authors:** Aili Yimamu, Heyin Yang, Wei Feng, Zunupiya Wufuli, Tilakezi Tuersun

**Affiliations:** a Department of Cardiology, The First People’s Hospital of Kashi Prefecture, Kashi, China.

**Keywords:** coronary heart disease, drug-eluting stents, in-stent restenosis, remnant cholesterol

## Abstract

In-stent restenosis (ISR) remains a major obstacle in percutaneous coronary intervention (PCI) in patients with coronary heart disease. Although previous studies suggest that remnant cholesterol (RC) may play a significant role in ISR, the exact relationship remains underexplored. This study aims to investigate the predictive value of RC levels for the risk of ISR following PCI and drug-eluting stent (DES) implantation. A retrospective study was conducted on 548 CHD patients who underwent PCI at the First Affiliated Hospital, Sun Yat-sen University, between January 2018 and December 2023. The primary outcome was the presence of ISR, defined as ≥50% in-segment diameter stenosis. Patients were divided into 2 groups: ISR (n = 56) and non-ISR (n = 492). The ISR group exhibited significantly higher RC levels compared to the non-ISR group (*P* = .004). Multivariable logistic regression analyses revealed that RC (odds ratio [OR] = 2.873, 95% CI: 1.121–7.364, *P* = .028), diabetes (OR = 2.656, 95% CI: 1.474–4.786, *P* = .001), and alcohol consumption (OR = 2.988, 95%CI: 1.082–8.251, *P* = .035) were independent predictors of ISR. The predictive logistic model combining RC with 6 clinically relevant factors (age, gender, smoking status, alcohol consumption, hypertension, and diabetes) for ISR showed a higher area under the curve of 0.721 (95% CI: 0.654–0.789), compared to the model with only RC, which had an area under the curve of 0.618 (95% CI: 0.547–0.690). Increased RC levels were significantly associated with ISR in CHD patients following PCI. This study suggests that RC could be a potential risk factor and predictive marker for the development of ISR after DES implantation.

## 1. Introduction

Coronary heart disease is a leading cause of global morbidity and mortality and remains the primary cause of death in China.^[1]^ According to the *China Cardiovascular Disease Report 2020* from the National Center for Cardiovascular Disease, among the 330 million individuals affected by cardiovascular diseases, 11.39 million have been diagnosed with CHD.^[2]^ Percutaneous coronary intervention (PCI) has become the primary strategy for coronary artery revascularization in patients with coronary artery disease. In 2018, the average incidence of PCI in China was 651 cases per million population, with an average of 1.46 stents placed per patient, and the mortality rate of coronary intervention was reported at 0.26%.^[2]^ The introduction and advancement of drug-eluting stents (DES) have significantly improved the efficacy and safety of PCI by markedly reducing the incidence of in-stent restenosis (ISR). However, despite the widespread use of DES, ISR continues to occur in approximately 5%–15% of patients, presenting a critical challenge in the management of CHD.^[3–5]^ The development of ISR is a progressive process driven by multiple pathophysiological mechanisms, with atherosclerosis playing a central role.^[6]^ As a result, identifying independent predictive factors for ISR and developing strategies to reduce its incidence have become essential research priorities to enhance clinical outcomes.

The development of ISR is a progressive process driven by multiple pathophysiological mechanisms, including systemic and procedural factors. Established systemic risk factors include diabetes mellitus, renal insufficiency, hypertension, and a history of coronary artery bypass grafting.^[7–10]^ Meanwhile, procedural determinants such as stent type, stent length, poor stent-vessel wall apposition, and perioperative complications like contrast-induced nephropathy have been consistently linked to higher ISR incidence.^[11,12]^

Beyond these systemic and procedural factors, circulating biomarkers, particularly lipid-related parameters, also serve as important risk factors for ISR. Low-density lipoprotein cholesterol (LDL-C) has long been recognized as a key risk factor for atherosclerotic cardiovascular disease and ISR. Nevertheless, numerous studies have shown that patients may still be at high risk for cardiovascular disease, even when LDL-C levels are within recommended ranges. To compensate for the limitations of LDL-C, multiple novel lipid parameters have been explored in recent years. The monocyte to high-density lipoprotein ratio has been found to be correlated with ST-segment elevation myocardial infarction patients treated with bare-metal stent implantation, which can effectively reflect vascular inflammation and endothelial repair status after coronary intervention.^[13]^ In addition, the non-high-density lipoprotein cholesterol to high-density lipoprotein cholesterol ratio is independently associated with the risk of ISR after DES implantation in patients with CHD and exhibits a superior predictive capacity for ISR compared to other lipid parameters.^[14]^ Although these lipid parameters have shown promising predictive performance, these biomarkers cannot fully explain residual restenosis risk.

Remnant cholesterol (RC) is derived from triglyceride-rich lipoproteins, including very-low-density lipoprotein and intermediate-density lipoprotein in the fasting state, and chylomicron remnants in the non-fasting state. The accumulation of RC in the arterial wall may contribute significantly to the development of atherosclerosis, similar to LDL-C.^[15]^ Emerging evidence has highlighted the potential role of RC as an independent risk factor for atherosclerosis and ISR.^[16–18]^ However, the role of RC in ISR remains underexplored, and further investigation is warranted. Therefore, this study aims to explore the relationship between preoperative RC levels and the occurrence of ISR in patients undergoing PCI. By identifying key risk factors and potential prognostic markers, this research seeks to improve risk stratification and contribute to better clinical management of ISR.

## 2. Methods

### 2.1. Study design and population

This retrospective, single-center study was conducted at the Cardiology Department of the First Affiliated Hospital, Sun Yat-sen University. Eligible patients who underwent PCI with DES implantation between January 1, 2018 and December 30, 2023, and completed the follow-up period were enrolled. The inclusion criteria were as follows: patients who underwent coronary angiography and DES implantation during hospitalization; patients who returned for follow-up coronary angiography 6 to 24 months post-procedure, including both inpatient and outpatient follow-up. The exclusion criteria were as follows: incomplete clinical data; chronic total occlusion in the vessel where the original stent was implanted or stent diameter <2.25 mm; 3) a history of severe hepatic or renal insufficiency, malignant tumors, or autoimmune rheumatic diseases.

All procedures were performed in accordance with the ethical standards laid down in the 1964 Declaration of Helsinki and its later amendments. This study was approved by the Ethics Committee of the First Affiliated Hospital of Sun Yat-sen University and was conducted in accordance with the local legislation and institutional guidelines for good clinical practice. Since this is a retrospective study, patient informed consent was waived.

### 2.2. Data collection

Baseline clinical data, including demographic information (age, gender), lifestyle factors (smoking and alcohol history), laboratory values (lipid profile, creatinine, and RC levels), past medical history (hypertension, diabetes), and medication history (including antiplatelet agents, ACE inhibitors/angiotensin receptor blockers angiotensin-converting enzyme inhibitor/angiotensin II receptor blocker, β-blockers, and statins), were extracted from the medical records prior to the first PCI. Imaging-related data, including the site of stent implantation, the number and length of stents, and the degree of stenosis at the original stent site during follow-up, were also collected.

Coronary atherosclerotic heart disease was defined as at least one of the following: coronary angiography-confirmed stenosis of ≥50% of the internal diameter of at least one subepicardial coronary artery or its major branches, a history of coronary revascularization, or a history of myocardial infarction. Subepicardial coronary arteries included the left main, left anterior descending, left circumflex, and right coronary arteries. The major branches included the first diagonal, second diagonal, obtuse marginal, rimmed, left ventricular posterior, and posterior descending branches. Hypertension was defined as systolic blood pressure ≥140 mm Hg and/or diastolic blood pressure ≥90 mm Hg without using antihypertensive medication, or having been treated with antihypertensive medication (≥1 year), or lower than 140 mm Hg/90 mm Hg with a previous history of hypertension, which could be ruled out as secondary to the hypertension by medical history and relevant investigations.^[19]^ Diabetes mellitus was defined as random venous plasma glucose ≥11.1 mmol/L or fasting glucose ≥7.0 mmol/L or 2-hour plasma glucose ≥11.1 mmol/L when 75 g of anhydrous glucose was dissolved in 300 ml of water and taken over 5 minutes.^[20]^

### 2.3. Coronary arteriography and interventional therapy

Coronary arteriography was performed using digital subtraction angiography (DSA, Allura Xper FD20 from Philips Healthcare, Eindhoven, The Netherlands) using Ultravist, Iodixanol and Visipaque contrast media. Right coronary angiogram was completed by left anterior oblique 50° or right anterior oblique 30° views. Left coronary angiogram was completed by right anterior oblique 50°, LAO 50°, cranial (CRA) 30°+LAO50° or caudal (CAU) 30°+LAO50° views. Evaluation of coronary arteriography findings and PCI for both groups was performed by the same experienced interventionalists. Dual antiplatelet therapy was prescribed for a minimum of 6 months after PCI, with the duration adjusted based on the individual patient’s bleeding risk and embolic risk.

### 2.4. Outcome

In this study, ISR was defined as the presence of ≥50% luminal stenosis within 5 mm of the stented segment, including both ends, as observed on follow-up coronary angiography.^[21]^ Based on follow-up coronary angiography results, patients were divided into 2 groups: ISR (in-stent restenosis) and non-ISR.

### 2.5. Residual lipid measurements and calculations

Total cholesterol, LDL-C, and high-density lipoprotein cholesterol were measured using venous blood from an 8-hour fasting blood draw collected from participants preoperatively on the morning of the second day of admission. LDL-C was determined directly by LDL Cholesterol Assay Kit (direct approach) on Beckman AU5800 analyzers. Residual lipid was calculated and estimated using the Friedewald formula (total total cholesterol minus LDL-C minus high-density lipoprotein cholesterol).

### 2.6. Statistical analysis

Continuous variables were expressed as mean ± standard deviation or median (interquartile range) according to the normality of the data, which was assessed using the Shapiro–Wilk test. The differences between the 2 groups were evaluated by Student *t* test or the Mann–Whitney *U* test, as appropriate. Categorical variables are presented as frequencies and percentages, with group differences assessed using the chi-square (*χ*^2^) test or Fisher’s exact test. Univariate logistic regression analyses were conducted to individually evaluate each factor associated with ISR. Factors found to be significant (*P* < .05) or clinically relevant (e.g., age, hypertension, smoking history, and alcohol consumption) were included in multivariable logistic regression models. Receiver operating characteristic curves were constructed to assess the predictive value of the final model, and the area under the curve (AUC) with 95% confidence intervals (CI) was calculated. Statistical analyses were performed using R Statistical Software (version 4.1.2). All statistical tests were two-sided, and *P < *.05 was considered statistically significant.

## 3. Results

### 3.1. Baseline characteristics of participants

**Figure 1.**
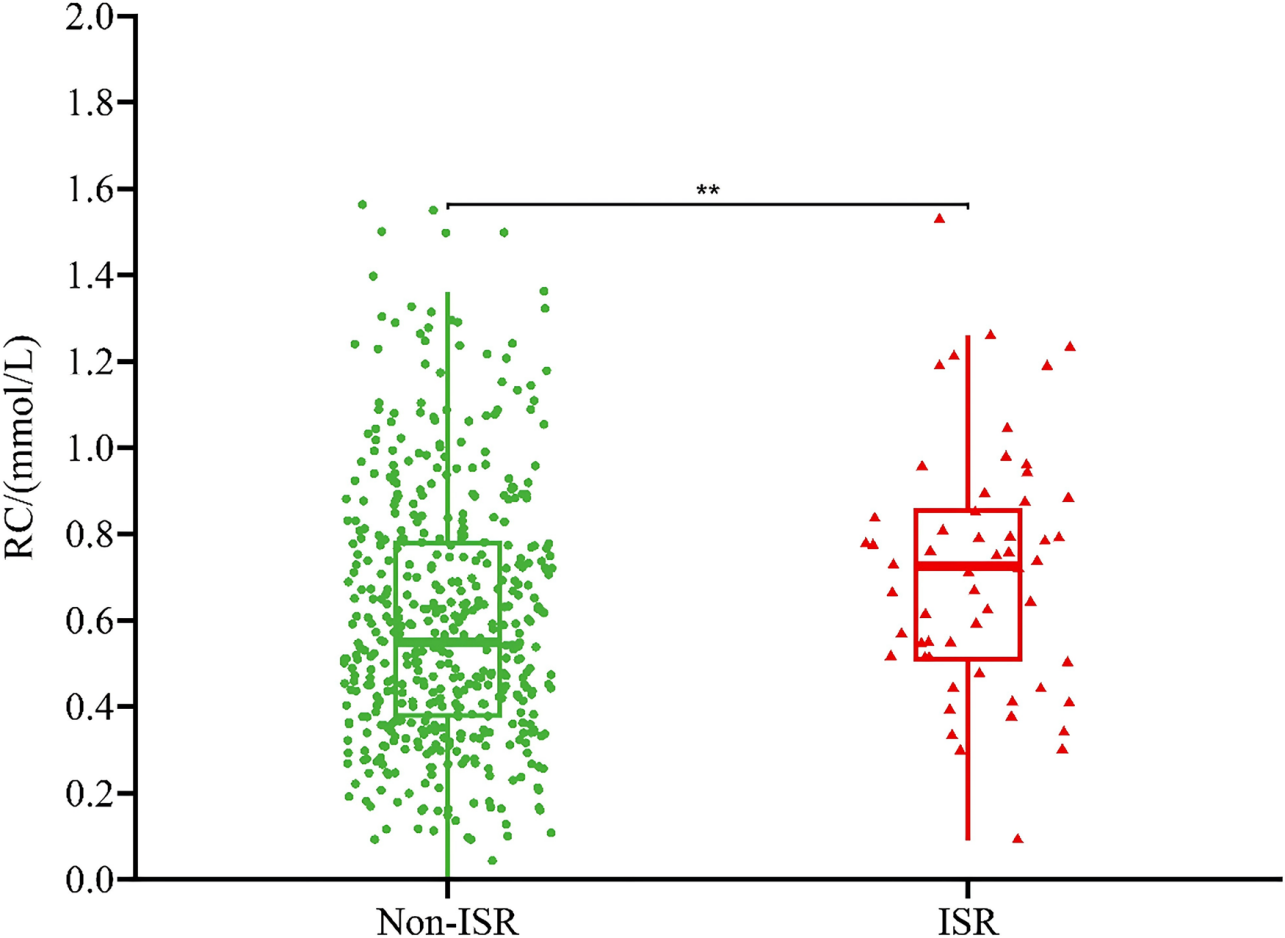
The differences in RC between the ISR group and the Non-ISR group. Statistically significant differences were indicated by: **: *P* < .01, RC = remnant cholesterol, ISR = in-stent restenosis.

A total of 548 patients were enrolled in the study, with 56 patients in the ISR group and 492 in the non-ISR group. Table 1 demonstrates the baseline characteristics of both groups. The median age was 62 (55, 68) for the ISR group and 64 (55, 72) for the non-ISR group (*P* = .201). The ISR group had a significantly higher proportion of females compared to the non-ISR group (*P* = .041). Additionally, the ISR group exhibited higher levels of LDL-C (*P* = .030) and RC (*P* = .004). The percentage of patients with a history of diabetes was also significantly higher in the ISR group (*P* = .001). Furthermore, the ISR group had a significantly longer stent length (*P* = .045) (Table 1 and Figure 1). Other baseline characteristics between the 2 groups showed no statistically significant difference.

**Table 1:** Baseline characteristics in restenosis and non-restenosis groups.

Characteristics	ISR group (n = 56)	Non-ISR group (n = 492)	*P*
Age, yr	62.00 (55.00, 68.00)	64.0 (55.50, 73.00)	.201
Female, n (%)	18 (32.1%)	99 (20.1%)	.041*
HDL-C, mmol/L	0.94 (0.85, 1.07)	0.97 (0.82, 1.13)	.580
LDL-C, mmol/L	2.94 (2.41, 3.69)	2.76 (2.18, 3.34)	.030*
TG, mmol/L	1.61 (1.24, 2.24)	1.50 (1.03, 2.17)	.580
Cr, umol/L	80.00 (66.00, 97.25)	82.00 (72.00, 96.00)	.405
length of stent, mm	40.50 (25.00, 68.75)	33.00 (18.00, 56.00)	.045*
RC, mmol/L	0.73 (0.51, 0.87)	0.55 (0.38, 0.78)	.004*
Diabetes, n (%)	29 (51.8%)	142 (28.9%)	.001*
Hypertension, n (%)	39 (69.6%)	310 (63.0%)	.380
Smoking status, n (%)			.580
Never smoking	32 (57.1%)	262 (53.3%)	
Former/current smoking	24 (3.57%)	230 (46.75%)	
Alcohol consumption, n (%)			.028*
Never drinking	51 (91.1%)	387 (78.7%)	
Former/current drinking	5 (0.00%)	105 (21.34%)	
Aspirin, n (%)	53 (94.6%)	468 (95.1%)	.999
Clopidogrel, n (%)	42 (75.0%)	410 (83.3%)	.137
Ticagrelor, n (%)	13 (23.2%)	96 (19.5%)	.596
Cilostazol, n (%)	0 (0.00%)	7 (1.42%)	.662
DAPT, n (%)	53 (94.6%)	473 (96.1%)	.716
Statin, n (%)	54 (96.4%)	477 (97.0%)	.689
ACEI/ARB, n (%)	44 (78.6%)	396 (80.5%)	.860
β-blocker, n (%)	54 (96.4%)	432 (87.8%)	.071
Number of implanted stents, n (%)			.080
1	19 (33.9%)	228 (46.3%)	
2	16 (28.6%)	143 (29.1%)	
>2	21 (37.5%)	121 (24.6%)	

Data are shown as medians (25th, 75th percentile) for continuous variables and as frequencies (percentages) for categorical variables.

ACEI = angiotensin-converting enzyme inhibitors, ARB = angiotensin II receptor blocker, Cr = creatinine, DAPT = dual antiplatelet therapy, HDL-C = high-density lipoprotein cholesterol, LDL-C = low-density lipoprotein cholesterol, RC = remnant cholesterol, TG = triglyceride.

### 3.2. Association between RC and risk of ISR

The results of the univariate logistic regression analysis are shown in Table 2. The analysis revealed that RC (odds ratio [OR] = 3.18, 95%CI: 1.324–7.635, *P* = .010), female sex (OR = 0.532, 95%CI: 0.291–0.972, *P* = .040), diabetes (OR = 2.647, 95%CI: 1.513–4.631, *P* = .001), length of implanted stent (OR = 1.008, 95%CI: 1.001–1.016, *P* = .033), and number of implanted stents (OR = 1.249, 95%CI: 1.021–1.527, *P* = .031) were significantly associated with ISR.

**Table 2:** Univariate logistic regression analysis of risk factors for in-stent restenosis.

Variables	OR	95%CI	*P*
RC	3.18	1.324–7.635	.010*
Female	0.532	0.291–0.972	.040*
Age, yr	1.022	0.994–1.05	.119
TG	1.216	0.945–1.564	.128
Hypertension	1.347	0.74–2.45	.329
Diabetes	2.647	1.513–4.631	.001*
Aspirin	0.906	0.264–3.11	.875
Clopidogrel	0.6	0.313–1.149	.123
Ticagrelor	1.247	0.645–2.411	.511
Cilostazol	0	0-Inf	.987
DAPT	0.71	0.203–2.478	.591
ACEI/ARB	0.889	0.452–1.748	.733
β-blocker	3.75	0.891–15.78	.071
Stent length	1.008	1.001–1.016	.033*
Stent numbers	1.249	1.021–1.527	.031*
LM	0.696	0.207–2.335	.557
LAD	1.371	0.754–2.493	.302
LCX	1.186	0.633–2.22	.594
RCA	1.504	0.864–2.617	.149
Smoking status (ref: nonsmoker)			
Former/Current smoking	0.614	0.287–1.313	.208
Alcohol consumption (ref: non-Alcohol)			
Former/ current drinking	2.497	0.892–6.990	.082
Cr	0.997	0.988–1.005	.434

Cr = creatinine, DAPT = dual antiplatelet therapy, LAD = left anterior descending artery, LCX = left circumflex artery, LM = left main coronary artery, RC = remnant cholesterol, RCA = right coronary artery, TG = triglyceride.

* *P*<0.05.

The results of the multivariable adjusted model are presented in Table 3. The results showed that RC (OR = 2.873, 95%CI: 1.121–7.364, *P* = .028) diabetes (OR = 2.656, 95%CI: 1.474–4.786, *P* = .001) and former or current alcohol consumption (OR = 2.988, 95%CI: 1.082–8.251, *P* = .035) were identified as independent risk factors for ISR.

**Table 3:** Multivariable logistic regression analysis of risk factors for in-stent restenosis.

Variables	OR (95%CI)	*P*
RC	2.873 (1.121, 7.364)	.028*
Female	0.581 (0.271, 1.246)	.163
Age, yr	1.025 (0.995, 1.057)	.104
Hypertension	1.147 (0.600, 2.193)	.678
Diabetes	2.656 (1.474, 4.786)	0.001*
Stent length	1.007 (0.991, 1.023)	.403
Stent numbers		
1	Ref.	.910
2	1.040 (0.465, 2.324)	.690
>2	1.300 (0.358, 4.720)	.675
Smoking status (ref: nonsmoker)		
Former/current smoking	0.531 (0.255, 1.104)	.090
Alcohol consumption (ref: non-Alcohol)		
Former/current drinking	2.988 (1.082, 8.251)	.035

RC = remnant cholesterol.

* *P*<0.05.

### 3.3. Predictive value for ISR

**Figure 2.**
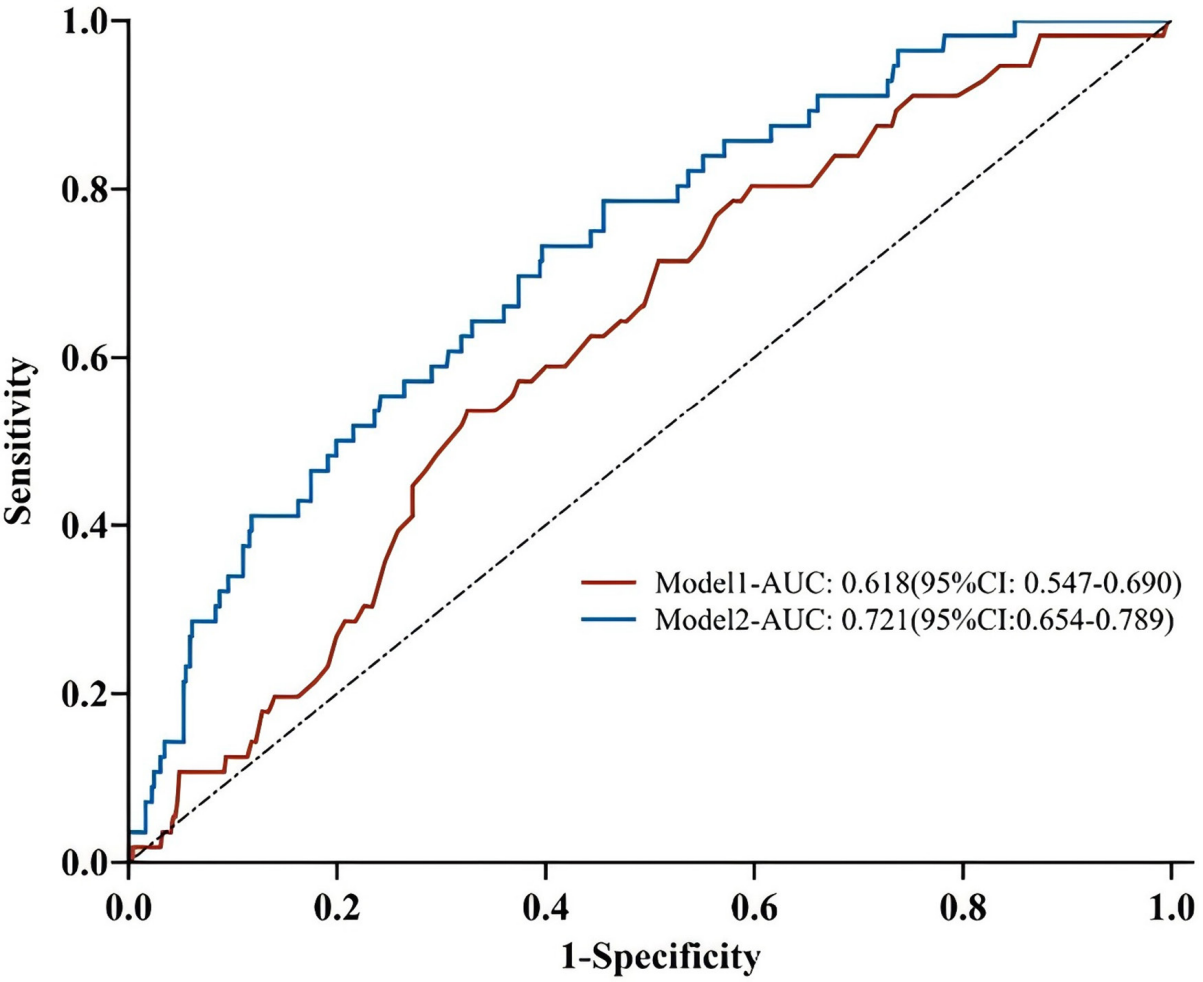
Area under the receiver operating characteristic curve of the 2 models in predicting in-stent restenosis. AUC = area under the curve, CI = confidence interval.

To assess the effectiveness of RC in the logistic regression model for predicting ISR, 3 logistic models were constructed. As demonstrated by the logistic regression, RC was significantly associated with ISR and was used as the sole predictor in Model 1, which demonstrated an AUC of 0.618 (95%CI: 0.547–0.690) (Fig. 2). Building upon Model 1, Model 2 further incorporated 6 clinically relevant risk factors: age, gender, smoking status, alcohol consumption, hypertension, and diabetes. This model achieved an AUC of 0.721 (95%CI: 0.654–0.789) (Fig. 2). The overall sensitivity of the 2 models was 0.536 and 0.732, respectively, while their specificity was 0.675 and 0.604, respectively. The optimal cutoff values are 0.685 and 0.0966, respectively.

## 4. Discussion

This retrospective study aimed to evaluate the predictive value of RC levels for ISR risk in patients with CHD following PCI. Our results showed that RC levels were significantly higher in the ISR group compared to the non-ISR group. Additionally, higher RC levels, diabetes, and current or past alcohol consumption were identified as independent predictors of ISR. These findings underscore the importance of RC as a potential risk factor for ISR.

Prior evidence has established that restenosis after PCI is influenced by both systemic and procedural factors,^[7–12]^ and lipid-related parameters such as monocyte to high-density lipoprotein ratio and non-high-density lipoprotein cholesterol to high-density lipoprotein cholesterol ratio have emerged as important prognostic markers for ISR.^[13,14]^ While traditional studies have focused on LDL-C as a primary risk factor for atherosclerosis and ISR ^[22–24]^, the role of RC in this setting remains incompletely understood, representing a significant gap for improving clinical management. Our findings directly address this gap, demonstrating that elevated RC is an independent predictor of ISR. This is supported by a growing body of evidence highlighting RC as a key contributor to residual cardiovascular risk, even in patients with optimal LDL-C levels.^[5,16–18]^ For instance, Elshazly et al analyzed 5754 patients across 10 interventional trials to assess whether RC correlates with coronary atheroma progression and adverse clinical events independent of LDL-C, and confirmed that RC levels independently predict coronary atherosclerosis.^[25]^ Furthermore, recent studies have specifically identified RC as an independent risk factor and a reliable predictor for ISR following PCI.^[16,26]^

The pathogenic mechanisms of RC remain poorly understood and are thought to include oxidative stress and inflammatory mechanisms. Similar to LDL-C, Anette Varbo proposed that RC may contribute to atherosclerosis by the mechanism that RC is readily oxidized in plasma and is the major oxidized lipoprotein in plasma.^[27]^ High levels of RC in serum increase penetration into the arterial wall compared with LDL-C, and RC is more readily captured and taken up by macrophages and smooth muscle cells. Because of its small size, it has ten times the binding capacity to macrophages and smooth muscle cells and carries 40 times the cholesterol content of LDL-C. Elevated levels of RC may be related to increased enrichment of TG in LDL-C particles, especially TG-rich, high-concentration LDL-C, which are small particles that remain in the circulation longer.^[28]^ In addition, RC can be transported through the endothelium, leading to faster foam cell formation and the selective retention of residual particles in the subendothelial space and their slow efflux at a slower rate relative to their rate of entry.^[29–31]^ Inflammatory mechanisms, on the 1 hand, allow RC to enter the arterial wall with triglycerides, triggering local or even systemic low-grade inflammation; residual blood lipoproteins can stimulate inflammatory factors in endothelial cells and promote monocyte adhesion.^[30]^ On the other hand, RC also induces the production of cytokines (tumor necrosis factor-a), interleukins (IL) (IL-1, IL-6, IL-8), and pro-atherosclerotic adhesion molecules, which activate inflammation and the coagulation cascade via fibrinogen activator inhibitors.^[32]^ Bernelot Moens et al indicated that high levels of RC may be related to inflammation of the arterial wall after endothelial injury.^[33]^ The persistence of inflammatory stimuli may lead to hyperproliferation of vascular smooth muscle cells and neoplastic endothelial proliferation, ultimately leading to ISR.^[34,35]^

In addition, the predictive model developed was able to more effectively predict ISR by RC and other clinical factors. A systematic review by Xiang et al summarized 22 existing ISR risk prediction models and reported that diabetes mellitus, number of diseased vessels, age, LDL-C and stent diameter were the most frequently adopted predictive variables.^[36]^ Although RC was not examined in that review, the conventional predictors they identified aligned with those in our study. Our findings directly address this gap: adding RC to 6 other clinical factors raised the AUC from 0.618 (95% CI: 0.547–0.690) for RC alone to 0.704 (95% CI: 0.547–0.776), and the combined model (Model 3) achieved an AUC of 0.721 (95% CI: 0.654–0.789). Notably, while RC inclusion improved sensitivity, it was accompanied by a reduction in specificity when combined with the other 6 factors. However, the overall improvement in AUC when RC was included demonstrates that RC can offer valuable prognostic information, which is in line with the findings of previous studies that emphasize the need for more comprehensive ISR prediction models. The development and validation of such integrative tools should draw on established methodological precedents, such as the risk prediction model recently proposed for myocardial injury in elderly patients undergoing nonelective surgery.^[37]^

The results of this study’s multivariate regression analysis also showed diabetes as an independent predictor of ISR. Although diabetes is a widely recognized risk factor for ISR as shown by a recent systematic review,^[36]^ in the context of the DES era, the role of diabetes as a significant predictor of ISR remains controversial.^[38]^ This variability in the literature might be explained by DES suppressing the differences in outcomes between diabetic and nondiabetic patients,^[39–41]^ and might be due to variability in various clinical variables, including the secondary prevention methods used as well as differences in the stent components. This is supported by a recent retrospective study that demonstrated significant differences in cardiovascular and in-stent stenosis and thrombosis outcomes between diabetic patients who utilized first-generation and second-generation DES.^[42]^

Our study results support that former/current alcohol consumption is an independent risk factor for ISR. The relationship between alcohol consumption and the rates of ISR remains conflicting in the literature; however, the majority of studies suggest an inverse relationship between alcohol consumption and adverse cardiovascular events, including ISR after PCI.^[43,44]^ The retrospective cohort study conducted by Zheng et al revealed that the degree of residual stenosis, stent diameter, and alcohol intake are independent predictors of ISR.^[45]^ Furthermore, studies suggest that the protective effect of alcohol consumption against ISR and cardiovascular disease is due to a reduction in the proliferative rate of cells in response to local stress and injury in the vessel walls.^[46]^ However, the relationship between alcohol and cardiovascular disease was found to be biphasic, with a direct correlation of outcomes on alcohol consumption being protective at low and moderate amounts and detrimental at high intakes.^[46]^ Therefore, a possible explanation for the differences between our study results and those reported in the literature is the limitation in not including subgroup analysis, including a lack of detailed information on participants’ drinking habits. The amount of alcohol consumed per day and the duration of their drinking history or periods of abstinence may have contributed to the observed discrepancies.

### 4.1. Limitations

Several limitations of this study should be acknowledged. First, the retrospective, single-center design with a relatively small sample size may introduce selection bias and unmeasured confounding, and increases the risk of Type II error while limiting the generalizability of our findings. Second, ISR was assessed visually by coronary angiography, which is inherently subjective; more objective modalities such as intravascular ultrasound or optical coherence tomography would have provided more precise measurements. Third, we lacked detailed data on perioperative procedural variables, including contrast volume, baseline renal function, and specific stent types or lengths: all of which have been previously associated with ISR risk after primary PCI. Additionally, variations in-stent design and deployment techniques may have influenced long-term patency rates, although these factors were not uniformly captured in our dataset. Fourth, lipid profiles were measured only at a single preoperative time point; serial measurements would have better captured dynamic changes in RC and other lipid parameters over time. Future prospective, multicenter studies with larger sample sizes, standardized procedural data collection, and objective imaging modalities are warranted to adjust for these confounders, externally validate our predictive model, and further elucidate the relationship between RC and ISR.

## 5. Conclusion

In patients with CHD who underwent PCI, the occurrence of ISR was significantly associated with elevated RC levels compared to non-ISR. This study suggests that RC may serve as a potential risk factor and predictive marker for ISR. However, further large-scale studies are needed to confirm the generalizability of these findings and to more clearly establish the causal relationship between RC and the development of ISR.

## Acknowledgments

Many thanks to all the teachers who guided this article and the colleagues who helped me.

## Author contributions

**Conceptualization:** Aili Yimamu, Tilakezi Tuersun.

**Formal analysis:** Aili Yimamu, Zunupiya Wufuli.

**Methodology:** Aili Yimamu, Wei Feng.

**Funding acquisition:** Tilakezi Tuersun.

**Writing – original draft:** Aili Yimamu, Tilakezi Tuersun.

**Writing – review & editing:** Heyin Yang, Tilakezi Tuersun.
